# Exosomal Surface Protein Detection with Quantum Dots and Immunomagnetic Capture for Cancer Detection

**DOI:** 10.3390/nano11071853

**Published:** 2021-07-18

**Authors:** Vojtech Vinduska, Caleb Edward Gallops, Ryan O’Connor, Yongmei Wang, Xiaohua Huang

**Affiliations:** Department of Chemistry, The University of Memphis, Memphis, TN 38152, USA; vvnduska@memphis.edu (V.V.); cgallops@memphis.edu (C.E.G.); rtconnor@memphis.edu (R.O.); ywang@memphis.edu (Y.W.)

**Keywords:** exosome, breast cancer, molecular detection, quantum dot, magnetic bead, fluorescent spectroscopy

## Abstract

Exosomes carry molecular contents reflective of parental cells and thereby hold great potential as a source of biomarkers for non-invasive cancer detection and monitoring. However, simple and rapid exosomal molecular detection remains challenging. Here, we report a facile method for exosome surface protein detection using quantum dot coupled with immunomagnetic capture and enrichment. In this method, exosomes were captured by magnetic beads based on CD81 protein expression. Surface protein markers of interest were recognized by primary antibody and then detected by secondary antibody-conjugated quantum dot with fluorescent spectroscopy. Validated by ELISA, our method can specifically detect different surface markers on exosomes from different cancer cell lines and differentiate cancer exosomes from normal exosomes. The clinical potential was demonstrated with pilot plasma samples using HER2-positive breast cancer as the disease model. The results show that exosomes from HER2-positive breast cancer patients exhibited a five times higher level of HER2 expression than healthy controls. Exosomal HER2 showed strong diagnostic power for HER2-positive patients, with the area under the curve of 0.969. This quantum dot-based exosome method is rapid (less than 5 h) and only requires microliters of diluted plasma without pre-purification, practical for routine use for basic vesicle research, and clinical applications.

## 1. Introduction

Extracellular vesicles (EVs) are membrane-bound vesicles that are released into extracellular environment by a spectrum of cell types [1]. EVs include three major subtypes, exosomes (EXOs), microvesicles (MVs), and apoptotic bodies, which are differentiated by size, biogenesis, molecular constitutes, and functions [2,3,4,5]. Intense research in the past decade has focused on EXOs due to their unique attributes [6]. EXOs are 30–200 nm in size and they are formed via exocytosis after fusion of multivesicular bodies with plasma membrane [7,8,9,10]. EXOs reflect both the nature of the parent cells as well as their pathophysiological state [11,12,13]. The molecular composition of EXOs consists of a variety of macromolecules including proteins, lipids, nucleic acids, and coding and non-coding RNA, which are reflective of a cell of their origin [7,14]. For instance, the cancer protein marker human epidermal growth factor 2 (HER2) is known to be overexpressed in primary cases of cancer [15,16,17]. Elevated levels of HER2 positive EXOs have been reported in serum samples from breast cancer patients in comparison to healthy donors [18]. Thus, biomarkers from tumor cells can be explored through exosomes.

EXOs enter blood and many other body fluids such as urine, saliva, ascites, and cerebrospinal fluid [19,20,21,22,23]. In addition, EXOs are fairly stable, tolerating multiple cycles of freezing and thawing, and can be preserved for years when stored in liquid nitrogen [24,25]. Thus, EXOs hold great potential as a source of biomarkers for developing new generation noninvasive liquid biopsy for cancer diagnostics and monitoring [3,4,26,27,28,29,30,31].

Detection of exosomal surface markers provides a powerful way for disease detection, as oncogenic receptors often reside within regions of the plasma membrane. Surface markers can be easily detected via target-specific labeling with optical contrast agents. For examples, Liang et.al. detected pancreatic cancer by detecting ephrin type-A receptor 2 on plasma exosomes with antibody-conjugated gold nanoparticles and dark field imaging [32]. In our previous research, we detected HER2-positive breast cancer using surface enhanced Raman scattering (SERS) nanoparticles to label and detect plasma exosomes after capture with target-specific antibodies onto a miniaturized device [33].

Among the different types of nanoparticle-based optical labels, quantum dots (QDs) have significant advantages for exosome detection. QDs are only 2–10 nm in diameter, which allows for efficient labeling and detection of exosomes at lower size range. QDs have strong fluorescence properties and superior photostability [34]. Monodisperse QDs with various functional ligands are readily accessible through commercial resources. Thus, QDs have attracted increasing interests for molecular exosome detection [35,36,37,38,39,40,41,42,43]. For example, Bai et al. used QDs to label and detect EXOs of different surface markers after trapping magnetic bead (MB)-isolated EXOs in a microfluidic micropillar chip [39]. Kim et al. developed a colorimetric-based lateral-flow assay to improve exosome detection using QD embedded in silica-encapsulated nanoparticles [43]. 

In this study, we demonstrate a simple yet effective method for EXO detection, using QDs in conjunction with immunomagnetic exosome isolation with MB targeting tetraspanin CD81 exosome markers. CD81 has been proven as a reliable marker for exosome identification [44]. CD81 expression is low on platelet cells, a major contributor of normal EXOs in plasma [45]. Thus, using CD81 capture ligands dramatically decreases the contamination of normal EXOs, enhancing the sensitivity of detecting tumor-derived EXOs. Although CD81 is not ubiquitously expressed on every exosome, CD81 expressions are common in different cancer EXOs [32,33,46]. We use MB conjugated with CD81 antibody for exosome capture to eliminate the need for long sample purification. For detection, we use highly fluorescent and universal QD655 linked with secondary antibodies to recognize primary antibodies that bind to targeted surface protein markers of interest on EXOs. This method was tested with different surface markers on EXOs from cell-derived EXOs in the breast cancer model. Using the method, we demonstrated that HER2-positive breast cancer can be diagnosed with exosome HER2 with high diagnostic power. Due to advantages in simplicity, speed, and low sample consumption, our QD-based method with magnetic separation holds strong promises for molecular detection of EXOs for basic vesicle research and clinical applications.

## 2. Materials and Methods

### 2.1. Materials

All reagents were purchased from Sigma-Aldrich (St. Louis, MO, USA) unless otherwise specified. Anti-rabbit CD81 antibody was purchased from Boster Biological Technology (Pleasanton, CA, USA). Antibodies targeting HER2, epidermal cell adhesion molecule (EpCAM), CD24, CD44, CD9, and CD63 were purchased from Biolegend (San Diego, CA, USA). Breast cancer cell lines SKBR3, MDA-MB 231, MCF7, and normal breast cell line MCF12A were purchased from ATCC (Manassas, VA, USA). PRMI and high glucose DMEM media were purchased from VWR (Radnor, PA, USA). NHS-activated MBs, QD655 with secondary antibodies, fetal bovine serum (FBS), and BCA kit were purchased from Fisher Scientific (Waltham, MA, USA).

### 2.2. Conjugation of Capture Antibody to MB

Prior to the conjugation, 20 µL of 10 mg/mL NHS-activated MBs were washed with 200 µL of ice-cold hydrochloric acid (1 mM) for 15 s. The beads were then collected using Qiagen 12-tube magnets and mixed with 200 µL of anti-rabbit CD81 antibodies (50 µg/mL) for 2 h at room temperature (RT). During the first 30 min, the mixture was vortexed every 5 min for 15 s. Then, the mixture was vortexed every 15 min for 15 s. After 2 h, CD81 antibody-conjugated MBs were collected with magnetic stand, the flow-through was saved for BCA analysis and the pellet was washed twice with 400 µL of Glycine (0.1 M, pH 2.0) and once with 400 µL of UP water. The CD81-MBs were then quenched for 2 h on a rotator at RT using 400 µL of Ethanolamine (3M, pH 9.0). At the end of the 2 h, CD81-MBs were collected and washed once with 400 µL of UP water followed by three consecutive washes with 400 µL of Dulbecco’s phosphate buffer solution (DPBS). 200 µL of DPBS with 0.05% sodium azide was then used as a storage buffer and CD81-MBs were stored at 4 °C until use. 

### 2.3. Collection and Purification of Cell-Derived EXOs

Breast carcinoma cells MDA-MB-231, MCF7, and SKBR3 were cultured in Dulbecco’s Modified Eagle Medium (DMEM) (MDA-MB-231, MCF7) and RPMI 1640 medium (SKBR3) supplemented with 10% FBS, 1% penicillin-streptomycin, 1% NEAA at 5% CO_2_ and 37 °C. Human breast normal cells MCF12A were cultured in DMEM: Nutrient Mixture F-12 with 5% fetal horse serum, 1% penicillin-streptomycin, 1% NEAA, 100 ng/mL cholera toxin, 0.5 mg/mL hydrocortisone, 10 µg/mL bovine insulin, and 20 ng/mL epidermal growth factor. When cells reached a confluency of approximately 70%, the medium was exchanged with serum-free medium and incubated for 48 h. Then, EXOs were isolated by differential centrifugation, as described previously [33]. Briefly, the culture medium was collected and centrifuged at 430 g for 10 min at RT. The supernatant was collected and centrifuged at 16,500× *g* for 30 min at 4 °C followed by 90 min centrifugation at 100,000× *g* at 4 °C. The supernatant was discarded, and the pellet was redispersed in ice-cold sterile DPBS, filtered with a 0.22 µm polyethersulfone (PES) filter (Millipore Express), and centrifuged again at 100,000× *g* for 90 min at 4 °C. The final exosome pellet was resuspended in 1 mL of ice-cold sterile DPBS and stored at −80 °C until use.

### 2.4. Source of EXOs from Patients and Human Donors

Plasma samples from human epidermal growth factor receptor 2 (HER2)-positive breast cancer patients were obtained from BioIVT (Westbury, NY, USA). Whole blood samples from different healthy donors were purchased Research Blood Components (Watertown, MA, USA). To obtain plasma from whole blood samples, whole blood was centrifuged at 2500× *g* for 15 min. The supernatant was collected and centrifuged again to obtain the plasma. Both collected and purchased plasma samples were diluted with sterile PBS and filtered with a 0.2 µm PES syringe filter (VWR) before use. The plasma and whole blood samples were not specifically collected for our research and we did not have access to the identifying information of the subjects. Based on U.S. Department of Health & Human Services regulations, our research is non-human subjects research.

### 2.5. Characterization of EXOs with Nanoparticle Tracking Analysis (NTA) and Scanning Electron Microscopy (SEM)

Plasma and cell-derived EXOs were characterized with NTA using a Nanosight LM10 microscope (Malvern Instruments, Inc, Westborough, MA, USA) to determine the concentration and size of EXOs. The samples were diluted to keep exosome concertation within the range of 10^6^ to 10^9^ EXOs per mL in accordance with the manufacturer’s recommendations. All samples were analyzed in triplicate of 40 s videos with camera level set at 12 and detection threshold set at 10. For SEM imaging, plasma exosomes from a HER2-positive breast cancer patient were fixed with 2% glutaraldehyde for 1 h at RT. Exosomes were then diluted with PBS and centrifuged at 100,000× *g* for 2 h. The pellet was resuspended in ultrapure water and a small amount (5 µL) was added onto a silicon chip and allowed to dry under a fume hood for 5 h, followed by further drying in a desiccator for overnight. Exosomes on the silica chip was coated with 2–3 nm gold film and then imaged with a Nova NanoSEM650 field emission SEM. Images were taken with voltage of 15 kV and magnification of 20,000×.

### 2.6. EXOs Capture and Fluorescent Detection

A total of 50 µL of EXOs at concentration of 1.0 × 10^9^/mL from breast cancer cells or human plasma were added to 10 µL of CD81 antibody-conjugated MBs (1 mg/mL) and mixed on a rotator for 1.5 h at RT. The MBs were then washed with DPBS and collected on the 12-tube magnets. The beads were resuspended with 50 µL PBS containing 2 ug/mL of target-specific antibodies. The mixture was mixed on a rotator for 2 h at RT and washed twice with sterile DPBS and magnetic separation. At last, the beads were resuspended with 50 µL of QD655 linked with secondary antibody (Invitrogen, 10 nM in BlockAid) and incubated on a rotator for 1 h at RT. After four instances of washing with DPBS and magnetic separation, the sample was resuspended in 50 µL of DPBS and transferred into a micro-quartz cuvette for fluorescence characterization. The fluorescence spectra were measured using a HITACHI F-2710 Fluorescence Spectrophotometer, with an excitation wavelength of 375.0 nm and emission from 600.0 to 700.0 nm. All the spectra were measured with the same instrumentation parameters including scanning speed (300 nm/min) and PMT voltage (400 V). A sample without a primary antibody was used as a negative control for background subtraction.

### 2.7. Enzyme-Linked Immunosorbent Assay (ELISA)

A total of 50 µL of cell-derived EXOs (1.0 × 10^9^/mL) were added into a 96-well polystyrene plate (Nunc MaxiSorp) and incubated at 4 °C overnight. Captured EXOs were washed 3 times with DPBS, followed by blocking with 200 µL of 1% BSA at RT for 2 h. EXOs were then washed three times with DPBS and treated sequentially with following solutions: 50 µL of 2 µg/mL of target-specific antibodies (2 h, RT), 50 µL of anti-mouse secondary antibody conjugated with horseradish peroxidase (HRP, 1:3400 dilution in 1% BSA, 2 h, RT), and 100 µL of 3,3′,5,5′-tetramethylbenzidine (TMB, 30 min, RT). Oxidation of TMB was stopped with 100 µL of 2 M sulfuric acid (H_2_SO_4_). Each step was followed by three washes with DPBS containing 0.1% tween 20 (DPBST), and two with DPBS. The optical density was measured at 450 nm using a BioTEK ELx800 microplate reader. DPBS without primary antibody was used as the negative control.

### 2.8. Micro BCA Assay

To confirm successful conjugation of CD81 antibodies to MBs, the unreacted antibodies were quantitatively measured using micro BCA assay. The assay was performed according to the standard manufacturer protocol. Briefly, 150 µL of Working Reagent (WR, 25:24:1 MA:MB:MC) was added into each well, together with 150 uL of each standard in a range from 0.0–1.0 µg/mL. The microplate was incubated at 37 °C for 2 h. Absorbance was measured with BioTEK ELx800 microplate reader at 540 nm.

### 2.9. Statistical Analysis

Statistical analysis was performed to compare the expression levels of targeted proteins on different cell lines and between cancer and healthy controls using analysis of variance (ANOVA) with the post hoc Scheffe method. A *p*-value ≤ 0.05 was considered significantly different. The mean difference between different groups was considered to be significant if the absolute value was greater than the minimum significant difference derived from the Scheffe method. The diagnostic value of HER2 in breast cancer patients was evaluated by receiver operation characteristic (ROC) curve analysis using R packages.

## 3. Results & Discussion

### 3.1. Design of the Methodology

**Figure 1:**
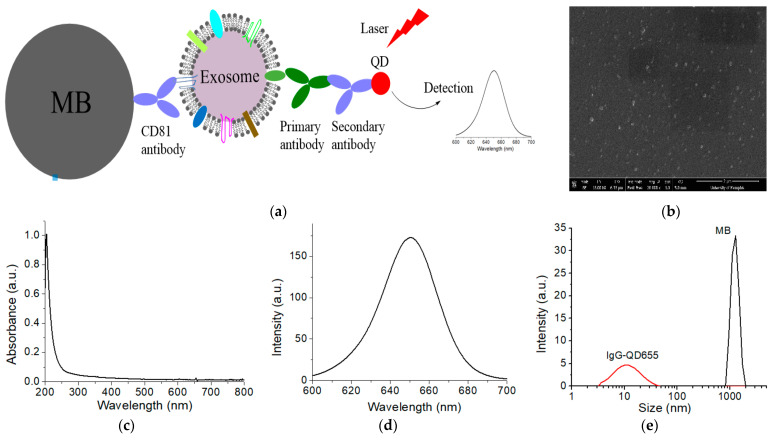
Schematic of the QD-based EXO assay (**a**), and characterization of EXOs (**b**), and QDs (**c**–**e**). EXOs were captured from biofluids with MB via CD81 monoclonal antibodies. Targeted surface cancer marker was recognized with primary antibody and then detected with secondary antibody-conjugated QD655. Signals were measured with fluorescence spectroscopy to quantify the QDs and correspondingly the surface protein markers on EXOs. (**b**) SEM image of plasma exosomes from a BC patient. (**c**) Absorption spectrum and (**d**) emission spectrum of IgG-QD655. (**e**) DLS characterization of the hydrodynamic size of IgG-QD655 and MB.

Figure 1a shows the schematic design of exosomal surface marker detection using QDs in conjunction with magnetic separation with immuno-MBs. The method involves three straightforward steps: (1) capturing cell-derived or plasma EXOs with anti-CD81 antibody-conjugated MBs; (2) labeling surface cancer markers of interest on the captured EXOs with specific primary antibodies; and (3) binding the secondary antibody-conjugated QD655 to the primary antibodies and detecting them using fluorescence spectroscopy. To facilitate comparison between different samples, we use EXOs of the same concentration (1.0 × 10^9^/mL). EXOs were pre-filtered with a 0.2 µm filter to remove cell debris or other impurities. Purity of exosomes were examined by NTA and SEM. NTA not only determines the size distribution of exosomes, but also measure the concentration. SEM can further examine the morphology of exosomes. Figure 1b shows the SEM of plasma EXOs from a HER2-positve breast cancer patient. The morphology (round shape) and size distribution (lower than 200 nm) of the particles under SEM confirm that they are EXOs. We would like to emphasize the EXOs we detected by QDs are much purer than those we characterized by NTA and SEM. This is as we use MBs linked with CD81 antibodies to further purify EXOs. Non-exosome vesicles, protein aggregates, and other cellular impurifies that were in the size range of EXOs were thus eliminated. The MBs also enrich EXOs to improve detection sensitivity.

To further improve detection sensitivity, we chose commercially available far red-fluorescent QD655 (emission peak around 655 nm) (Figure 1c,d). This QD655 is one of the brightest QDs, with quantum yield of 0.6. In particular, the size of QDs is small, usually 2–10 nm. Even after conjugation with secondary antibody, the mean hydrodynamic size is only approximately 12 nm (Figure 1e). For signal readout, a regular fluorescence spectrometer is used to measure the exosome solution in a microliter cuvette (50 µL). This capture, labeling, and detection method is extremely simple and easy to operate, practical for use in research and clinic labs.

### 3.2. Characterization of the Specificity and Sensitivity

**Figure 2:**
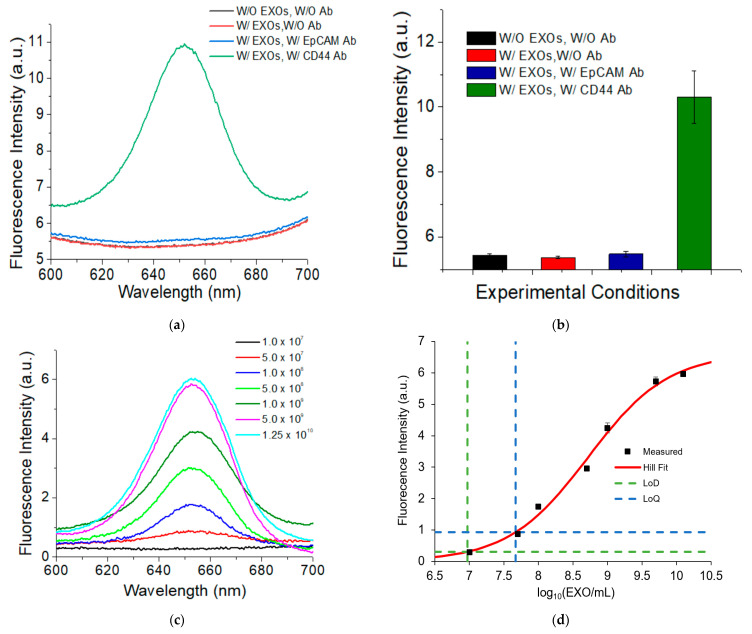
Examination of the specificity (**a**,**b**) and sensitivity (**c**,**d**) of the QD-based EXO assay. (**a**) Fluorescence spectra of MDA-MB-231 EXOs treated under four different conditions. (**b**) Intensity plot of (**a**) using the fluorescence intensity at 655 nm. (**c**) Fluorescence spectra of SKBR3 EXOs targeting HER2 at different concentrations. (**d**) Dose–response curve based on data in (**c**) using the fluorescence intensity at 655 nm. Signals were background corrected using the signals without the presence of HER2 primary antibody. Error bar is the standard deviation from triplicate experiments. Ab: antibody.

Using MDA-MB-231 EXOs as the model, we examined the specificity of the QD-based EXO assay. Figure 2a shows the fluorescence spectrum of EXOs targeting high expression marker CD44 (olive), in comparison to three controls: EXOs targeting negative marker EpCAM (blue), absence of CD44 primary antibody (red), and absence of both EXOs and CD44 primary antibody (black). Figure 2b shows the intensity plot of Figure 2a.

The results show that a strong fluorescence peak from QD655 was observed for EXOs targeting CD44 whereas fluorescence signals from QD655 were not detected for the three negative controls, suggesting the high specificity of our QD-based EXO assay. The three controls gave similar background signals that were most likely due to the scattering of the MBs and EXOs as well as instrumental noise.

Using SKBR3 EXOs as the model, we examined the sensitivity of our assay using high expression HER2 as the protein marker. A series of dilutions of SKBR3 EXOs were made to determine the limit of detection (LOD). Figure 2c shows a mean of fluorescence spectra (*n* = 3) of different EXO concentrations and Figure 2d shows the dose–response curve of data from the fluorescence peak at 655 nm. All data were background corrected using the signals at 655 nm without the presence of a HER2 primary antibody. The results showed that the intensity of QD655 fluorescence signals increased with the increase in the EXO concentration. Signals reach saturation after 1.0 × 10^10^ EXOs/mL. The studies showed that the LOD was 9.3 × 10^6^ EXOs/mL. The concentration of exosomes in human plasma is >10^9^/mL. Thus, our assay can detect exosomes at a concentration at least 107 times lower than a typical concentration of exosomes in plasma. This sensitivity was achieved using an excitation wavelength of 375 nm, scanning speed of 300 nm per min, and voltage at 400 V. The limit of quantification (LOQ) was determined to be 4.7 × 10^7^ EXOs/mL. The working concertation for the rest of the studies was set to 1.0 × 10^9^ EXOs/mL.

### 3.3. Validation with ELISA

**Figure 3:**
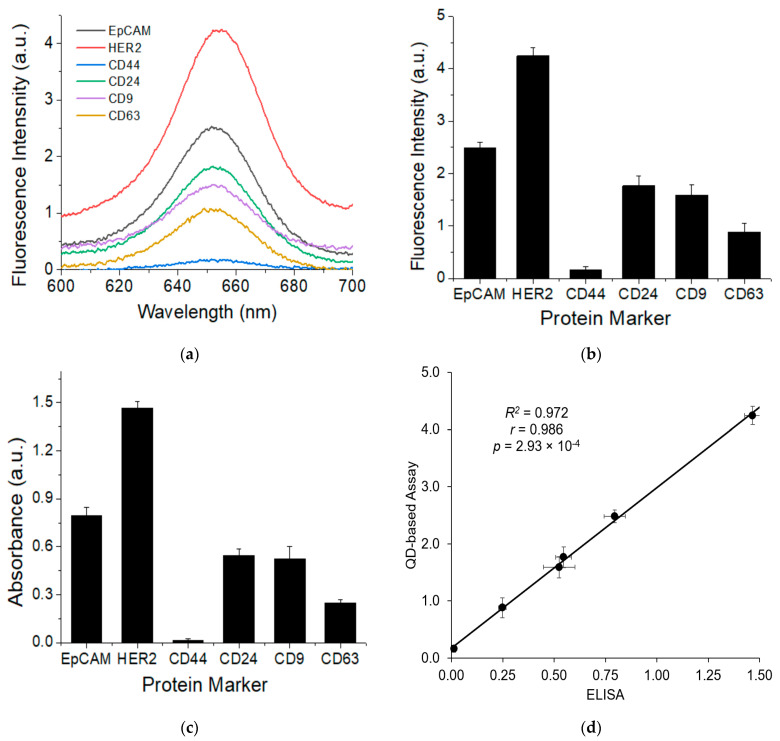
Comparison of QD-based assay and ELISA for the detection of exosome surface protein markers. (**a**) Fluorescence spectra of targeted markers on the surface of SKBR3 EXOs using the QD-based method. (**b**) Protein expression profile based on data in (**a**) at the mean intensity of 655 nm. (**c**) Protein expression profile of targeted surface markers on the surface of SKBR3 EXOs determined using ELISA. **(d**) Comparison of QD-based assay with ELISA for measurement of protein expressions on exosomes. Error bar is the standard deviation from triplicate experiments.

To validate our assay for the detection of surface proteins on EXOs, we analyzed six different surface markers on SKBR3 EXOs and compared the results with those using ELISA (Figure 3). The six proteins were categorized into three different groups: epithelial marker EpCAM, breast cancer markers HER2, CD24, and CD44, and exosome markers CD9 and CD63. These markers have a varied expression on the SKBR3 cells, with high expression of HER2, moderate to high EpCAM and CD24, and low expression of CD44 [47,48,49].

Figure 3a shows the mean fluorescence spectra of all the markers (*n* = 3) and Figure 3b shows the expression profile of these markers on the SKBR3 EXOs based on the data from Figure 3a. The results show that SKBR3 EXOs have a high expression HER2 marker, moderate to high expression of CD9, CD63, CD24, and EpCAM, and very low expression of CD44 marker. These results are consistent with our previous reports using a Raman-based assay [33].

The measured expressions of the six markers on SKBR3 EXOs with ELISA were shown in Figure 3c,d. ELISA was performed in an indirect mode, in which EXOs were adsorbed onto a 96-well plate, labeled with primary antibodies, and detected with HRP-conjugated secondary antibodies. Similar to our QD-based method, the ELISA results showed high expression HER2, moderate to high expression of CD9, CD63, CD24, and EpCAM, and very low expression of CD44. A side-by-side comparison (Figure 3d) shows a strong correlation of the two methods, with a correlation coefficient of 0.972. Compared to ELISA, our QD-based assay is much quicker, with a turnaround time of 4.5 h in contrast to 2 days for ELISA.

### 3.4. Detection of Different Protein Markers on EXOs Derived from Different Cell Lines

**Figure 4:**
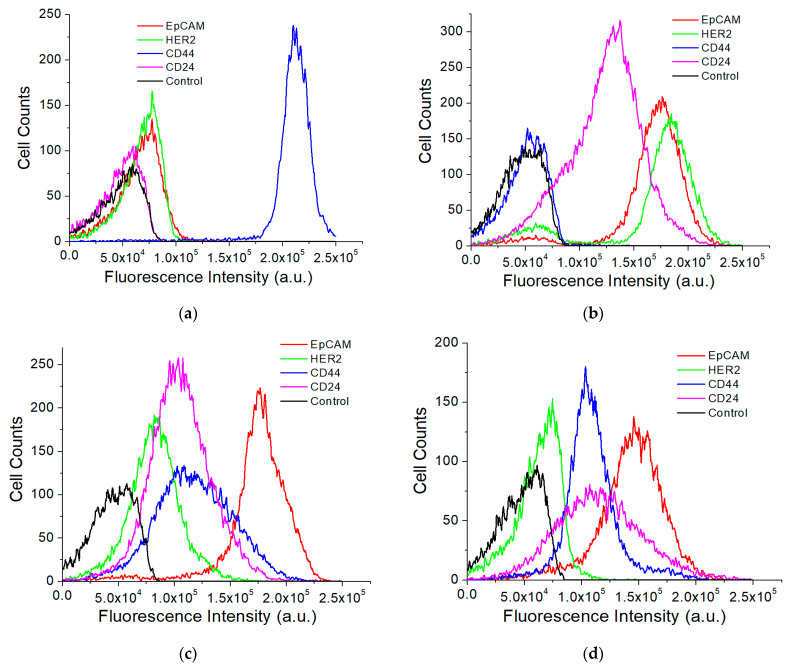
Flow cytometry analysis of different cell surface protein markers for MDA-MB-231 (**a**), SKBR3 (**b**), and MCF7 (**c**) breast cancer cell lines and normal breast cell line MCF12A (**d**).

Using the QD-based assay, we compared the expression of four common breast cancer-associated surface protein markers EpCAM, HER2, CD44, and CD24 on three breast cancer cell lines, SKBR3, MDA-MB-231, and MCF7, and compared with those from a normal breast cell line MCF12A. Flow cytometry shows distinct expression patterns of these markers on these cell lines (Figure 4). SKBR3 is a HER2-positive breast cancer cell line. It also has a strong expression of EpCAM and moderate expression of CD24. MDA-MB-231 is a triple-negative metastatic breast cancer cell line. It is known to have extremely high expression of CD44, but not CD24. The MCF cells have strong expression of EpCAM and moderate expression of CD44 and CD24. The MCF12A normal cells have a weak expression of EpCAM, HER2, CD44, and CD24.

**Figure 5:**
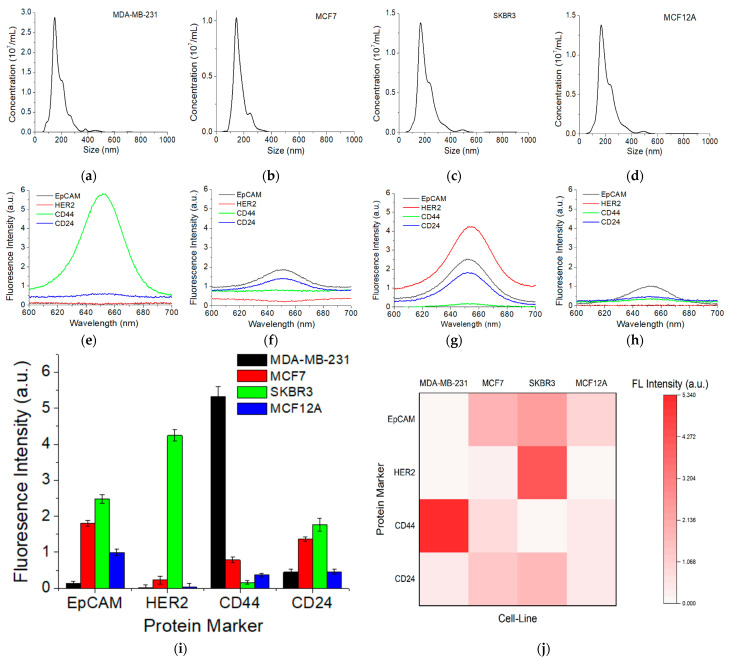
Detection of surface protein markers on EXOs derived from different breast cancer cells (MDA-MB-231, MCF7, and SKBR3) in comparison to normal cells (MCF12A). (**a**–**d**) Size distribution of EXOs measured with NTA. (**e**–**h**) Fluorescence spectra of EXOs targeting different surface markers. (**i**) Comparison of protein marker expressions based on the fluorescence mean intensity at 655 nm. The *p*-values among the four cell-lines for markers EpCAM, HER2, CD44, and CD24 are 2.3 × 10^−8^, 6.2 × 10^−10^, 1.3 × 10^−9^, and 3.0 × 10^−6^, respectively. (**j**) Heatmap comparison of protein expression based on data in (**i**). Error bar is the standard deviation from triplicate experiments.

NTA characterization (Figure 5a–d) shows the size of EXOs derived from SKBR3, MDA-MB-231, MCF7, and MCF12A was 167 ± 80, 149 ± 65, 145 ± 45, and 138 ± 71 nm, respectively. Figure 5e–h shows fluorescence spectra of each marker on EXOs derived from each cell line. Signals were corrected with the background, the fluorescence spectrum without primary antibody. Figure 5i is a quantitative comparison of the background-corrected fluorescence intensity at 655 nm. Figure 5j is a heatmap comparison using the data from Figure 5i. Compared to the protein expression of these markers on cells (Figure 4), it is clear that EXOs reflect the surface protein expression of their originating cells. For example, CD44 is highly expressed on MDA-MB-231 EXOs. HER2 is highly expressed on SKBR3 EXOs but it is negative on MDA-MB-231 EXOs and MCF12A normal exosomes. It has a low expression on MCF7 exosomes. Expression of EpCAM follows a decreased order of SKBR3, MCF7, MCF12A, and MDA-MB-231. The normal MCF12A EXOs are negative for HER2, but have low expression of CD44 and CD24.

### 3.5. Detection of Breast Cancer Via Plasma EXOs

**Figure 6:**
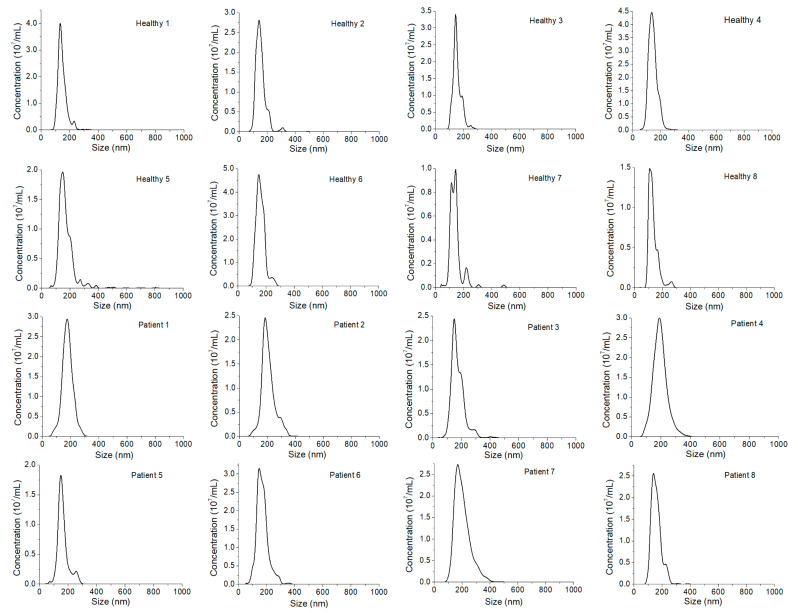
Size distribution of EXOs in plasma from different HER2-positive breast cancer patients (Patient 1 to Patient 8) and different healthy donors (Healthy 1 to Healthy 8) measured by NTA.

The clinical potential of our QD-based EXO method for cancer diagnostics was evaluated using HER2-positive breast cancer as the disease model. HER2-positive BC is one of the major BC subtypes. Identification of HER2 overexpression directs effective treatment with trastuzumab. For proof-of-concept studies, we analyzed plasma from eight HER2-positive breast cancer patients and eight healthy donors. All plasma samples in this study were diluted with 1x PBS, and filtered through a 0.2 µm PES filter. No further purification was performed. Figure 6 shows the size of plasma samples from all the human subjects. The size of EXOs ranged from 107 ± 35 to 189 ± 48 nm, without statistically significant differences between healthy donors and cancer patients. Based on the concentrations determined by NTA, all samples were further diluted to a concentration of 1.00 × 10^9^ EXOs/mL before use.

**Figure 7:**
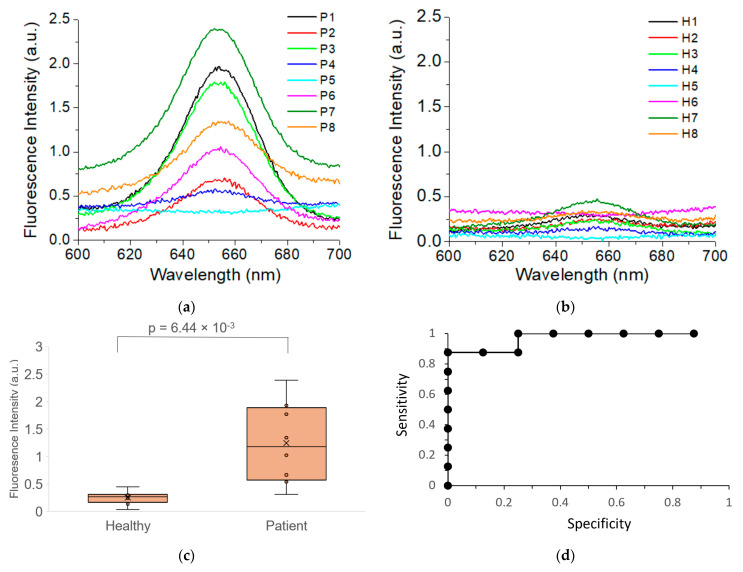
Detection of HER2-positive breast cancer using QD-based EXO assay. (**a**) Average fluorescence spectra from triplicate experiments of HER2-targeted EXOs from each patient with HER2-positive breast cancer. (**b**) Average fluorescence spectra from triplicate experiments of HER2-targeted EXOs from each healthy donor. (**c**) Comparison of the exosomal HER2 expression between patient (*n* = 8) and healthy control (*n* = 8). (**d**) Roc curve for detecting HER2-positive breast cancer by QD-based EXO assay.

Figure 7a,b show the fluorescence spectrum of EXOs from patients (a) and healthy controls (b). The results showed that six cancer patients (75%) showed fluorescence signals from 0.5 to 2.4 while all eight healthy controls gave signals lower than 0.45. Statistical analysis with ANOVA showed that the mean fluorescence intensity of HER2 expression on the plasma EXOs from the cancer patients was significantly different from that of healthy control, with a *p*-value of 6.44 × 10^−3^ (Figure 7c). The HER2 expression was approximately five times higher than that of healthy control (1.24 ± 0.74 for patient versus 0.25 ± 0.12 for healthy control). Further ROC analysis with sensitivity and specificity showed that exosomal HER2 expression was a strong diagnostic marker for HER-positive patients, with AUC = 0.96875. This is consistent with previous studies by our group using the Raman method [33] and other groups using surface plasm resonance [50]. The studies showed our QD-based exosome assay can detect breast cancer via HER2 detection and quantification using plasma exosomes from patients.

## 4. Conclusions

In conclusion, we have demonstrated that exosomal surface markers can be quantitatively detected using QDs in conjunction with magnetic separation with microbeads. In this method, exosomes were captured and concentrated onto magnetic microbeads, followed by recognition of targeted surface markers with primary antibody, and then detection with secondary antibody-conjugated QDs. Using this QD-based method, we can specifically and quantitatively detect different surface protein markers on exosomes from different breast cancer cell lines. We can also differentiate cancer exosomes from normal exosomes using cancer-associated surface protein markers. Using pilot clinical samples, we have shown that HER2-positive breast cancer can be detected by analysis of HER2 expression on plasma exosomes using QDs in conjunction with magnetic separation and enrichment. Cancer patients show about five times higher HER2 expression than healthy donors. The high AUC value (AUC = 0.96875) suggests exosomal HER2 as a strong diagnostic marker for HER2-positive patients.

Compared to previous methods using QDs, our method is simple and rapid. Our method follows a straightforward capture, labeling, and measurement methodology, requiring only ~45 h total time. It does not require extensive pre-preparation of plasma samples and sophisticated instrumentation. Detection was simply performed with bulk fluorescence measurement with a routine fluorescence spectrometer. The method is also highly sensitive, with LOD of 9.3 × 10^6^ EXOs/mL that is over 100 times lower than a typical exosome concentration in plasma. Due to the advantages in simplicity, high sensitivity, and widely accessible instrumentation, our method can be widely used in research and clinical laboratory.

We would like to point out that our research results need to be further validated with a larger cohort before clinical applications. However, we believe the sample size in this study is sufficient for proof-of-concept studies. As demonstrated in Figure 7, a post hoc analysis of the data had a power level >95%. Additionally, we would like to point out that the throughput at the current stage is also limited. However, the method can be adapted for simultaneous analysis of multiple samples using multi-well magnetic microplates such as the EpiMag HT magnetic separator. For signal readout, a portable fluorescence spectrometer may be used to measure samples directly in the microplates in the absence of the magnets.

## Data Availability

The data presented in this study are available on request from the corresponding author.
